# Longitudinal Associations Between Household Solid Fuel Use and Handgrip Strength in Middle-Aged and Older Chinese Individuals: The China Health and Retirement Longitudinal Study

**DOI:** 10.3389/fpubh.2022.881759

**Published:** 2022-06-30

**Authors:** Yashu Liu, Qing Chang, Yang Xia, Yuhong Zhao

**Affiliations:** Department of Clinical Epidemiology, Shengjing Hospital of China Medical University, Shenyang, China

**Keywords:** cohort, clean fuel, handgrip strength, solid fuel, household air pollution

## Abstract

**Background:**

Household solid fuel have been associated with changes of handgrip strength (HGS). However, no study has explored the longitudinal associations between household solid fuel use and HGS. Thus, the aim of our cohort study was to investigate the longitudinal associations between household fuel use and HGS.

**Methods:**

The study was based on the China Health and Retirement Longitudinal Study. A handheld dynamometer was used to measure HGS. Household fuel use statuses were collected using questionnaires. Analyses of covariance were performed to examine the associations between household fuel use and HGS.

**Results:**

The study included 9,382 participants during a 4-year follow-up. The participants who used solid fuel for cooking had more decreases of HGS than those who used clean fuel (*P* < 0.0001). The least square means (95% CIs) of changes of HGS for participants who used solid fuel and those who used clean fuel for cooking were −1.67 (−2.15, −1.19) and−2.27 (−2.75, −1.79), respectively. The association between fuel use for heating and HGS was non-significant (*P* = 0.63). The interaction terms of sex to cooking fuel (*P* = 0.04) and smoking to cooking fuel (*P* < 0.001) were significant; men and participants who had ever smoked had higher decreases in HGS.

**Conclusion:**

Using household solid fuel for cooking but not heating was associated with more decreases in HGS. Proper ventilation and clean fuel should be promoted for public health.

## Introduction

Age-related reductions in muscle mass and strength begin in middle age and potentiate near the fifth or sixth decade of life (1, 2). One of the main indicators of upper body muscle strength is handgrip strength (HGS) (3). HGS is sensitive to age-related changes and changes in biological function and is not only an indicator of muscle strength but also of biological vitality (4, 5). Studies published in the past few years confirmed that HGS was associated with mortality, length of hospital stay, physical functioning, cancer, cognitive function, and depression (1, 6–8). Moreover, HGS is a non-invasive and simple marker of skeletal muscle strength and hand function that is recommended in clinical setting and epidemiology studies (8, 9). Thus, HGS measurement is appealing as a quick and inexpensive way to stratify an individual's risk of diseases, especially in older adults.

Indoor air pollution is responsible for up to 4% of the burden of disease in low-income countries and is considered a major public health problem globally (10). A previous study suggested that, although the use of electricity and gases had increased, more than 40% of the Chinese use solid fuel as a household energy source, which results in household air pollution (11, 12). The incomplete combustion of solid fuel releases particulate matter (PM), heavy metals (such as arsenic and lead), and other pollutants (13). A previous study found that higher concentrations of lead in blood were associated with reduced gait speed and weakness (14). Another study found that long-term exposure to PM2.5 (fine inhalable particles, with diameters that are generally 2.5 micrometers and smaller) was associated with decreases of skeletal muscle mass and increases of body fat mass among healthy elderly persons living in Taipei Basin (15). It is reasonable to hypothesize that exposure to solid fuel use is also associated with low HGS. To the best of our knowledge, only one cross-sectional study has explored the association between the solid fuel use and HGS in low- and middle-income countries. The results suggested that greater use of solid fuel was associated with low HGS (16). However, considering the cross-sectional design of the study, large and prospective cohort studies are needed. Moreover, the main purposes for using solid fuel are cooking and heating, and they have different exposure patterns (17). However, to the best of our knowledge, the evidence of the associations between different purposes of household solid fuel use and HGS is limited.

Thus, we conducted this large cohort study to investigate the prospective associations between different purposes of solid fuel use and HGS in middle-aged and older Chinese individuals.

## Methods

### Participants

This cohort study used data from the China Health and Retirement Longitudinal Study (CHARLS) and included approximately 10,000 households and 17,708 individuals in 150 counties/districts and 450 villages/resident communities of China. A previous study had described the details of this cohort study (18). CHARLS included three datasets, namely, the baseline dataset from 2011 to 2012, the first follow-up dataset from 2013 to 2014, and the third follow-up dataset from 2015 to 2016.

**Figure 1:**
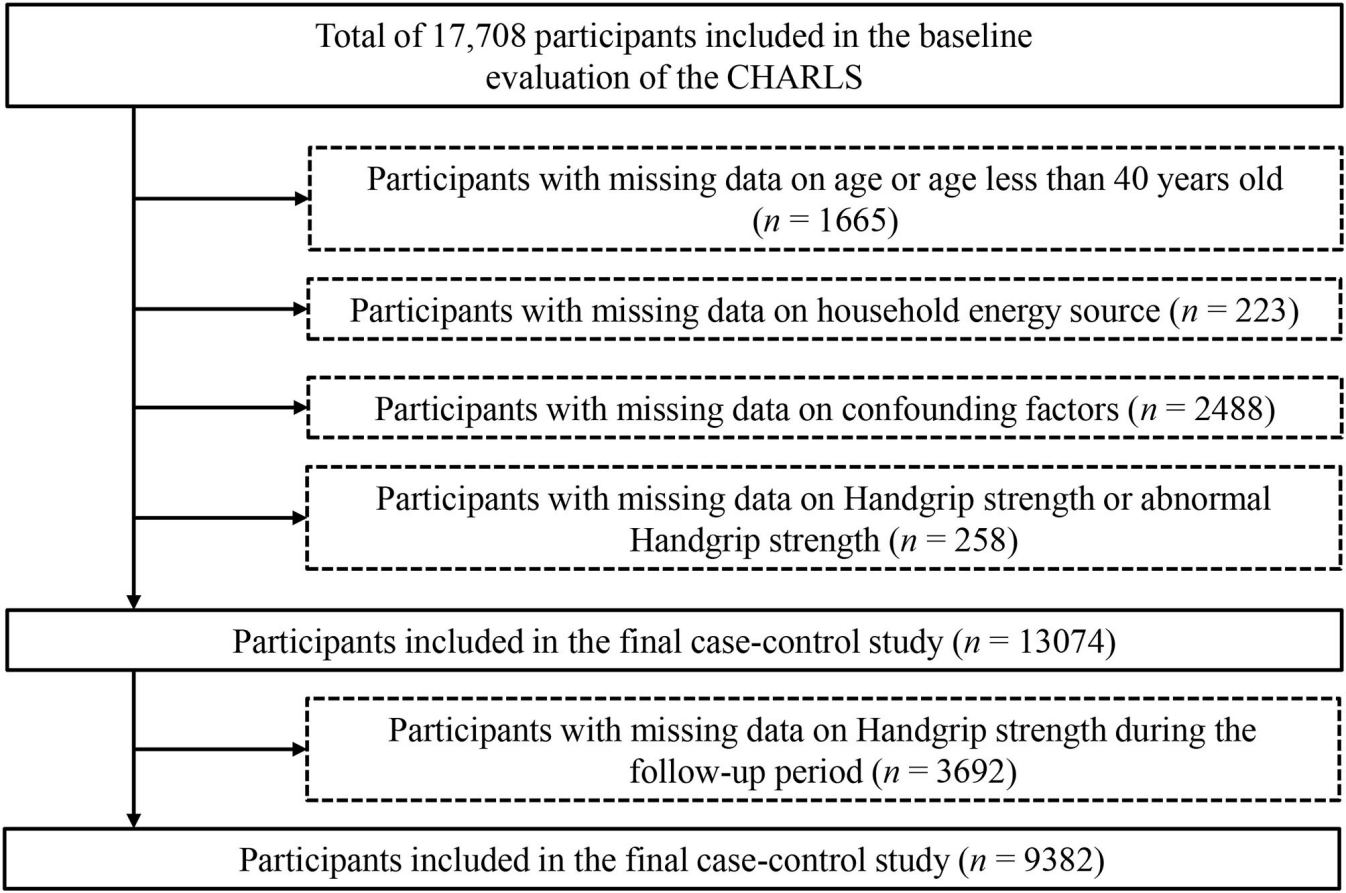
Flow chart of the selection process of participants. CHARLS, China Health and Retirement Longitudinal Study.

The study involved 17,708 Chinese people in the baseline investigation from 2011 to 2012. We excluded participants who had missing age data or those <40 years old (*n* = 1,665), did not have the baseline information of household energy source (*n* = 223) or confounding factors (*n* = 2,488), and had abnormal (0 kg or more than 100 kg) or missing HGS values (*n* = 258). Furthermore, we excluded another 3,692 participants lost to follow-up or had missing or abnormal values of HGS during the 4 years follow-up. Finally, the study included 9,382 participants. The inclusion and exclusion process of this study's participants is depicted in Figure 1.

### HGS

Participants who did not undergo any surgical procedures, suffer from disease, injury, or severe pain in either hand during the 6 months prior to the study were asked to undergo HGS evaluation (kilogram, kg). HGS was evaluated by trained technicians using a hand-held dynamometer (YuejianTM WL-1000, Nantong Yuejian Physical Measurement Instrument Co., Ltd., Nantong, China). The participants were in a standing position with their arms hanging when their HGS was tested, and they were asked to squeeze the handles of the dynamometer as hard as possible. Each hand was tested twice. The maximum HGS from the four tests in both hands of each participant was used for analysis.

### Household Energy Sources

Questionnaires were used to assess household energy sources (cooking and heating fuel). Cooking fuel included natural gas, marsh gas, liquefied petroleum gas, electric coal, crop residue, wood, biomass charcoal, and others. Heating fuel included natural gas, solar energy, liquefied petroleum gas, electric, municipal heating, coal, crop residue, wood, biomass charcoal, and others. In this study, natural gas, marsh gas, solar energy, liquefied petroleum gas, electric, and municipal heating were regarded as clean fuel; coal, crop residue, wood, biomass charcoal, were regarded as solid fuel. Furthermore, the “others” was an option chosen by the participants who did not need to cook or heat their dwellings. Thus, we excluded the participants who chose the “others” option to perform sensitivity analysis.

### Confounding Factors

Trained interviewers collected the baseline demographic and confounding factors using questionnaires according to the standard procedure (including age, gender, educational status, smoking, drinking, marriage status, residence, income status, social activities, number of chronic diseases, and retirements). The PM2.5 data was obtained from the data center in the National Aeronautics and Space Administration Earth Observing System Data and Information System (19). The mean values of the city-level PM2.5 concentrations from 2011 to 2015 were calculated and included in the analysis as a confounding factor. The detailed information of assessment and the definition of confounding factors are described in Supplementary Material.

### Statistical Analysis

Baseline participant characteristics were described according to household fuel use: clean fuel users (both heating and cooking), solid fuel users for one purpose (either heating or cooking), and solid fuel users for two purposes (both heating and cooking). Analysis of variance (ANOVA) or chi-square test was used to explore the differences in baseline characteristics. Continuous and categorical variables are presented as least square means (95% confidence intervals [CIs]) and percentages, respectively. A covariance analysis was used to estimate the associations between household fuel use for different purposes and changes of HGS. Bonferroni analysis was used to compare differences among groups. The changes of HGS were defined as HGS in 2015 minus HGS in 2011. Four stepwise models adjusted for either known risk factors or sociodemographic factors were used: Crude model was without any adjustment; Model 1 adjusted for age, gender, and body mass index; Model 2 further adjusted for educational status, smoking, drinking, marriage status, residence, income status, social activities, number of chronic diseases, and retirements and baseline HGS; Model 3 further adjusted ambient concentration of PM2.5. When solid fuel use for cooking and heating and the changes of HGS were explored separately the other purpose of fuel use was adjusted for in Model 3.

Moreover, subgroup analyses were performed according to sex (men or women) and smoking status (never-smokers or ever-smokers). The interaction effects between household fuel use and sex/smoking on HGS were tested. The multiplicative term of household fuel use and sex or household fuel use and smoking status in all adjustments of confounding factors were calculated to tested the significances of interaction effects. In addition, the participants who did not explicitly choose types of fuel were excluded in order to perform sensitive analysis. The sensitive analysis of association between household fuel use and relative HGS (HGS/ BMI) was performed. Statistical Analysis System 9.4 edition for Windows (SAS Institute Inc., Cary, NC, USA) was used to analysis all data. Differences were considered statistically significant when *P*-values <0.05 (two-tailed).

## Results

### Participants' Characteristics

The baseline characteristics of participants categorized by types of fuel use are presented in Table 1. Totally, 9,382 participants were included in the study. Near half (44.53, 45.78, and 46.62%) were male with a mean age of 57.25, 57.33 and 59.16 years old in clean fuel users (both heating and cooking), solid fuel users (either heating or cooking) and solid fuel users (both heating and cooking), respectively. Solid fuel users (both heating and cooking) tended to be older (*P* < 0.0001) and reside in rural villages (*P* < 0.0001). Moreover, solid fuel users (both heating and cooking) had lower levels of education (*P* < 0.0001) and income (*P* < 0.0001). In addition, the smoking rates (*P* < 0.0001) and the chronic disease numbers (*P* < 0.0001) of those who used solid fuel for both heating and cooking were significantly higher. Those who used clean fuel for both heating and cooking tended to be retired (*P* < 0.0001) and attended social activities (*P* < 0.0001).

**Table 1:** Baseline participant characteristics according to fuel users.

**Baseline characteristics**	**Clean fuel users (both heating and cooking)**	**Solid fuel users (either heating or cooking)**	**Solid fuel users (both heating and cooking)**	***P-*value^**a**^**
	***n* = 1,720**	***n* = 2,512**	***n* = 5,150**	
Handgrip strength in baseline (2,011 kg)	34.12 (33.64, 34.60)^b^	33.39 (33.00, 33.79)	31.77 (31.49, 32.05)	<0.0001
Handgrip strength in follow-up (2,015 kg)	31.57 (31.12, 32.02)	31.22 (30.85, 31.60)	29.20 (28.94, 29.46)	<0.0001
Age (years)	57.25 (56.82, 57.68)	57.33 (56.98, 57.69)	59.16 (58.92, 59.41)	<0.0001
Sex (male, %)	44.53	45.78	46.62	0.13
Body mass index (kg/m^2^)	24.43 (24.25, 24.61)	23.70 (23.55, 23.85)	23.17 (23.06, 23.27)	<0.0001
Marital status (current married, %)	87.67	89.97	88.02	0.68
Residence in rural village (yes, %)	35.99	59.83	81.03	<0.0001
Income (≥ mean value)	24.13	18.83	10.12	<0.0001
Participation in social activities (yes, %)	58.37	50.04	42.19	<0.0001
Retired (yes, %)	38.37	23.93	16.68	<0.0001
Educational level				
No formal education	12.09	19.31	26.39	<0.0001
Primary school	31.22	36.74	39.17	<0.0001
Middle school or above	56.69	43.95	34.45	<0.0001
Smoking status (%)				
Non-smoker	65.35	62.14	59.55	<0.0001
Ex-smoker	8.20	7.88	8.50	0.53
Smoker^c^	26.45	29.98	31.94	<0.0001
Drinking status (%)				
≥ 1 time/month	24.24	26.35	23.84	0.32
<1 time/month	9.36	7.60	7.28	<0.01
Never	66.40	66.04	68.87	0.02
No. of chronic diseases (%)				
0	34.07	33.88	31.63	0.03
1	32.85	30.77	29.34	<0.01
≥ 2	33.08	35.35	39.03	<0.0001

a *Analysis of variance or chi-square test*.

b *Least square mean (95% confidence interval) (all such values)*.

### Different Purposes of Solid Fuel Use and HGS

**Figure 2:**
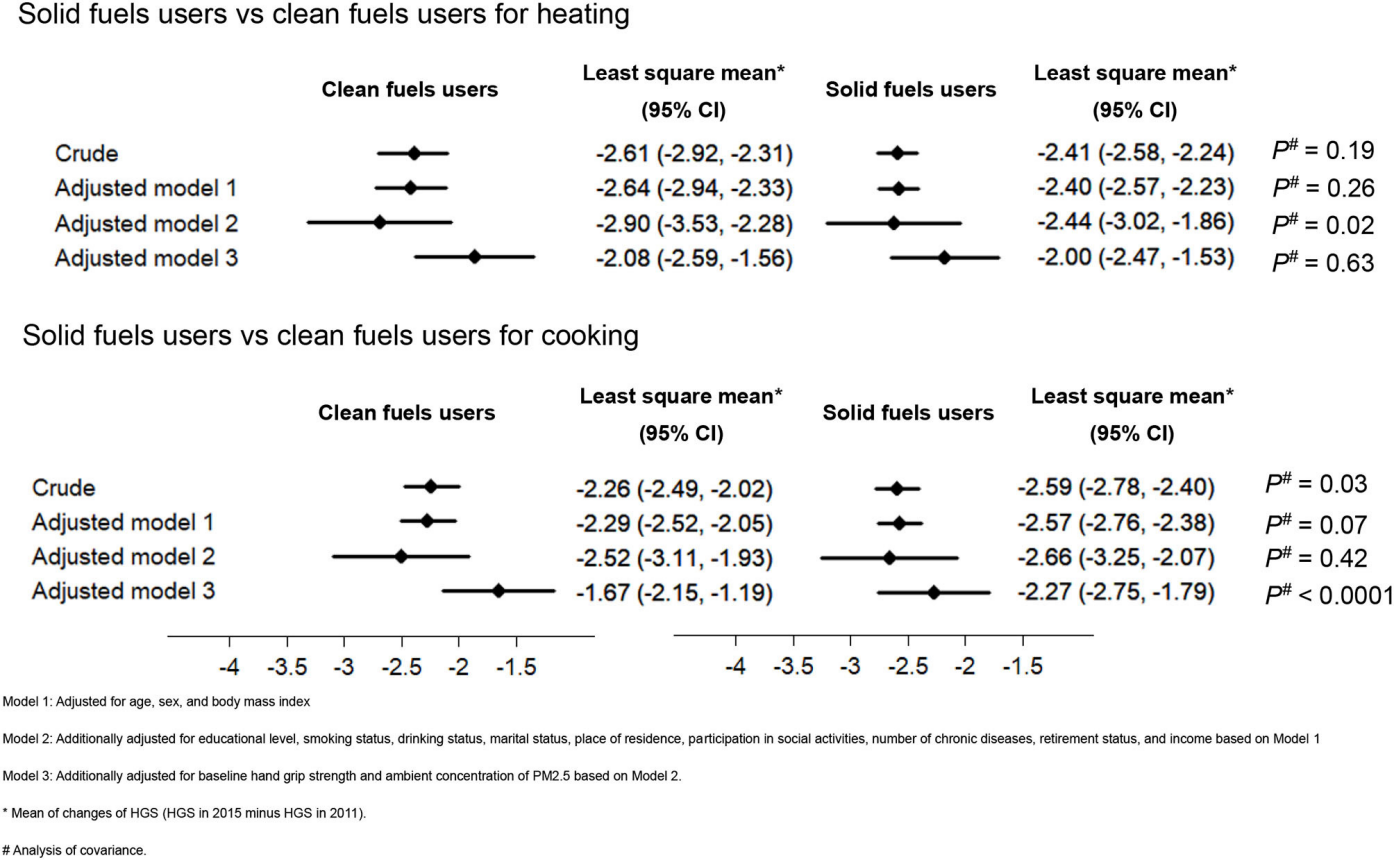
Associations between different purposes of solid fuel use and changes of HGS. HGS, handgrip strength.

Figure 2 presents the associations between the different purposes of household fuel use (cooking and heating) and HGS. For cooking, compared with solid fuel users, the HGS of clean fuel users were decreased significantly in the crude model (*P* = 0.03). The association remained significantly in the all-adjusted model (*P* < 0.001). However, for heating, there was no significant difference in the decreases in HGS during the follow-up between clean fuel and solid fuel users.

### Switching Types of Fuel and HGS

Considering that the data of heating fuel use status was limited in 2015, we explored whether switching types of fuel for cooking was associated with HGS (Table 2). Participants with missing data of cooking energy source during follow-up were excluded. Eventually, 5,796 participants were included. In the crude model, differences were non-significant between persistent solid fuel users, those who switched from solid fuel to clean fuel, those who switched from clean fuel to solid fuel, and persistent clean fuel users. The least square mean (95% CI) of changes of HGS in the aforementioned groups were −2.71 (−3.02, −2.41),−2.61 (−3.05, −2.17),−2.60 (−3.41, −1.79), and −2.34 (−2.67, −2.00), respectively. As shown in the all-adjusted model, compared with persistent solid fuel users (least square mean [95% CI] of changes of HGS = −2.59 [−3.21, −1.97]), persistent clean fuel users (least square mean [95% CI] of changes of HGS = −1.91 [−2.52, −1.31]) during the follow-up had significant decreases of HGS (*P* < 0.01).

**Table 2:** Association between switching types of fuel for cooking during the follow-up and changes of HGS^a^^*^.

	**Persisting solid fuel users**	**Switching solid fuel to clean fuel users**	**Switching clean fuel to solid fuel users**	**Persisting clean fuel users**
No. of participants	2,355	1,117	330	1,994
Crude	−2.71 (−3.02, −2.41)^b^	−2.61 (−3.05, −2.17)	−2.60 (−3.41, −1.79)	−2.34 (−2.67, −2.00)
Adjusted model 1^c^	−2.70 (−3.00, −2.39)	−2.61 (−3.05, −2.17)	−2.59 (−3.04, −1.78)	−2.36 (−2.70, −2.03)
Adjusted model 2^d^	−2.42 (−3.19, −1.64)	−2.36 (−3.18, −1.54)	−2.39 (−3.44, −1.33)	−2.34 (−3.09, −1.59)
Adjusted model 3^e^	−2.59 (−3.21, −1.97)	−2.36 (−3.02, −1.70)	−2.19 (−3.04, −1.70)	–**1.91 (**–**2.52**, –**1.31)**^**f**^

*HGS, handgrip strength*.

* *Changes of HGS: HGS in 2015 minus HGS in 2011*.

a *Analysis of covariance*.

b *Least square mean (95% confidence interval) (all such values)*.

c *Adjusted for age, sex, and body mass index*.

d *Additionally adjusted for educational level, smoking status, drinking status, marital status, place of residence, participation in social activities, number of chronic diseases, retirement status, and income based on Model 1*.

e *Additionally adjusted for baseline hand grip strength and ambient concentration of PM_2.5_ based on Model 2*.

f *P < 0.05, compared with solid fuel in both baseline and follow-up*.

### Subgroup Analysis and Sensitivity Analyses

Subgroup analyses were performed according to sex and smoking status (Table 3). For cooking, there were no significant differences between those who used clean fuel and solid fuel for cooking among men (*P* = 0.27), women (*P* = 0.07) and never-smokers (*P* = 0.53) in the crude model. However, these associations were significant after the adjustments for confounding factors, and the *P*-values were <0.01, <0.01, and 0.02, respectively. As shown, more solid fuel use for cooking was associated with more decreases of HGS in the ever-smokers, both in the crude model (*P* = 0.02) and after adjustment for confounding factors (*P* < 0.001). The differences of decreases of HGS between clean fuel users and solid fuel users were higher in ever-smokers (difference among groups (95% CI), 0.94 [0.46, 1.41]) than in never-smokers (difference among groups [95% CI], 0.41 [0.07, 0.74]). The interaction effects of fuel-sex and fuel-smoking status were explored (Table 3). The interaction terms of cooking fuel-sex (*P* for interaction <0.05) and cooking fuel-smoking status (*P* for interaction <0.001) were both significant after adjustments. However, the interaction terms of heating fuel-sex (*P* for interaction = 0.56) and heating fuel-smoking status (*P* for interaction = 0.20) were non-significant after adjustments.

**Table 3:** Association between heating and cooking solid fuel use and changes of HGS according to sex and smoking status ^*^.

	**Clean fuel users for cooking**	**Solid fuel users for cooking**	**P^a^**	***P* for interaction^**a**^**	**Clean fuel users for heating**	**Solid fuel users for heating**	**P^a^**	***P* for interaction^**a**^**
**Sex**								
Men				0.04				0.56
No. of participants	1,690	2,627			992	3,325		
Crude	−2.73 (−3.10, −2.37)^b^	−3.00 (−3.29, −2.71)	0.27		−3.17 (−3.65, −2.70)	−2.81 (−3.07, −2.55)	0.19	
Adjusted model 1^c^	−2.75 (−3.11, −2.38)	−2.99 (−3.28, −2.70)	0.31		−3.18 (−3.65, −2.70)	−2.81 (−3.07, −2.55)	0.19	
Adjusted model 2^d^	−2.95 (−3.64, −2.26)	−3.56 (−4.25, −2.86)	<0.01		−3.32 (−4.06, −2.57)	−3.24 (−3.91, −2.65)	0.75	
Adjusted model 3^e^	−2.95 (−3.64, −2.25)	−3.58 (−4.28, −2.89)	<0.01		−3.55 (-4.31, −2.78)	−3.26 (−3.93, −2.58)	0.26	
**Women**								
No. of participants	2,049	3,016			1,221	3,844		
Crude	−1.86 (−2.17, −1.56)	−2.24 (−2.49, −1.98)	0.07		−2.16 (−2.56, −1.76)	−2.06 (−2.29, −1.84)	0.68	
Adjusted model 1^c^	−1.89 (−2.20, −1.58)	−2.22 (−2.47, −1.96)	0.03		−2.18 (−2.58, −1.78)	−2.06 (−2.28, −1.83)	0.61	
Adjusted model 2^d^	−2.09 (−2.93, −1.25)	−2.63 (−3.47, −1.78)	<0.001		−2.19 (-3.05, −1.32)	−2.42 (−3.26, −1.59)	0.22	
Adjusted model 3^e^	−2.06 (−2.90, −1.22)	−2.65 (−3.49, −1.80)	<0.01		−2.32 (−3.19, −1.45)	−2.43 (−3.26, −1.59)	0.61	
**Smoking status**								
Never-smoker				<0.001				0.20
No. of participants	2,392	3,360			1,417	4,335		
Crude	−2.15 (-2.44, −1.86)	−2.27 (-2.52, −2.03)	0.53		−2.40 (−2.77, −2.02)	−2.16 (−2.38, −1.95)	0.29	
Adjusted model 1^c^	−2.16 (−2.46, −1.87)	−2.26 (−2.50, −2.01)	0.62		−2.40 (−2.78, −2.03)	−2.16 (−2.38, −1.95)	0.27	
Adjusted model 2^d^	0.28 (−0.29, 0.85)	−0.10 (−0.67, 0.47)	0.02		0.19 (−0.42, 0.79)	0.05 (−0.51, 0.62)	0.48	
Adjusted model 3^e^	0.29 (−0.28, 0.86)	−0.12 (−0.69, 0.46)	0.02		0.09 (−0.53, 0.71)	0.52 (−0.51, 0.61)	0.85	
**Ever-smoker**								
No. of participants	1,347	2,283			796	2,834		
Crude	−2.45 (−2.86, −2.05)	−3.06 (−3.37, −2.75)	0.02		−3.00 (-3.53,−2.47)	−2.79 (-3.07,−2.51)	0.49	
Adjusted model 1^c^	−2.45 (−2.86, −2.04)	−3.06 (−3.37, −2.75)	0.02		−3.01 (−3.54, −2.48)	−2.79 (−3.07, −2.51)	0.47	
Adjusted model 2^d^	−4.44 (-5.22, −3.65)	−5.32 (−6.10, −4.54)	<0.001		−4.88 (−5.72, −4.04)	−4.90 (−5.66, −4.14)	0.95	
Adjusted model 3^e^	−4.41 (−5.20, −3.62)	−5.35 (−6.13, −4.57)	<0.001		−5.20 (−6.06, −4.34)	−4.94 (−5.70, −4.17)	0.35	

*HGS, handgrip strength*.

*Changes of HGS: HGS in 2015 minus HGS in 2011.

a *Analysis of covariance*.

b *Least square mean (95% confidence interval) (all such values)*.

c *Adjusted for age, sex, and body mass index*.

d *Additionally adjusted for educational level, smoking status, drinking status, marital status, place of residence, participation in social activities, number of chronic diseases, retirement status and income based on Model 1*.

e *Additionally adjusted for baseline hand grip strength and ambient concentration of PM_2.5_ based on Model 2*.

The participants who did not choose definite types of fuel were excluded in order to perform sensitivity analyses. The analysis of the association between the types of fuel use for heating and HGS included 7,682 participants; the analysis of the association between types of fuel use for cooking and HGS included 9,312 participants; the analysis of the association between household fuel use and relative HGS included 9,290 participants. All sensitivity analyses had no influence on the overall results.

## Discussion

This prospective cohort study found that the participants who cooked with solid fuel had more decreased HGS than those who cooked with clean fuel during a 4-year follow-up in middle-aged and older Chinese individuals. Moreover, we explored the association between switching types of fuel for cooking during the follow-up and the subsequent changes of HGS; the results demonstrated that the participants who persistently used solid fuel had more decreases of HGS than those who persistently used clean fuel. In addition, the results revealed that ever-smokers had more decreases in HGS than never-smokers.

The contribution of indoor solid fuel combustion to household air pollution is one of the leading environmental risk factors for many diseases and premature deaths (20). A previous study suggested that participant exposures to PM2.5, black carbon, and carbon monoxide from biomass cookstoves were double, four times, and twenty times higher than those from electric cookstoves, respectively (21). Another study found that long-term exposure to PM2.5 was associated with decreases of skeletal muscle mass and increases of body fat mass among healthy elderly persons living in Taipei (15). Thus, it is important and necessary to explore the associations between household fuel use and HGS.

There was one previous cross-sectional study which included 31,209 participants and explored the associations between household fuel use and HGS. The results of the previous study suggested that compared with clean fuel users, solid fuel users had relatively lower levels of HGS (β = −0.86 [95% CI: −1.35, −0.37]) after adjustments for confounding factors (16). Considering the different patterns of fuel usage for use cooking and heating (12), the household fuel use was categorized by household fuel for cooking and heating in our study. The results suggested that solid fuel use for cooking but not heating was associated with more decreases of HGS. Regarding solid fuel, cooking fuel were used more frequently and daily than heating fuel; moreover, heating activities are only performed in cold seasons and areas. Therefore, compared with solid fuel use for heating, the biological effects of solid fuel use for cooking were more significant. Although, there were differences between the previous study and our study, such as population, region and race, our results are in line with those of the previous study (16). Moreover, another study identified the same associations, which published in Chinese (22). In addition, no significant decreases of HGS were found among those who switched from clean fuel to solid fuel or switched from solid fuel to clean fuel during the four-year follow-up. The period after the switch may have been too short for the associations to be evident as well as the reason our results were not significant.

In the subgroup analysis, the associations between solid fuel for cooking and HGS were significant in different subgroups categorized by gender and smoking status. The main results were confirmed by the results of subgroup analysis. Moreover, we found that higher decreases in HGS were found among ever-smokers. The interaction effect between household fuel use for cooking and smoking on HGS was found. In the same way, a previous study found that there were additional interactions between solid fuel use and smoking for both cardiovascular and all-cause mortality (23). However, there were significant interaction effects of fuel-smoking status, for which the mechanism is unclear. More studies that investigate the interaction effects among types of fuel and smoking status should be performed. Moreover, the interaction terms of cooking fuel-sex were also significant after adjustments. Thus, the association between household fuel use and HGS could be impacted by the modifying effects of sex.

The specific mechanisms that associate solid fuel use and HGS are unclear, although there are several plausible biological explanations for the association. First, the fine PM released by the combustion of solid fuel is a chronic source of neuroinflammation and reactive oxygen species that contribute to neuropathology and central nervous system diseases (24). In addition, there can be damage to the nervous system because some smaller components of PM can reach the brain (25) and may lead to a decrease of neurotransmitter, which were associated with poor muscle function (26). Thus, household solid fuel use was associated with low-level HGS. Second, it is now clear that many inflammatory factors (such as tumor necrosis factor-α and interleukin-6) directly result in muscle degradation (27). Air pollution exposure is associated with systemic inflammation (28). Therefore, household air pollution may be associated with low HGS due to an inflammatory response.

This study is a prospective study to investigate the associations between household fuel use and HGS for different purposes and the prospective study design made it possible to study the causal associations between household fuel use and HGS. Furthermore, we are the first to explore the effect of switching types of cooking fuel from solid to clean upon declines in HGS. Nevertheless, we should not ignore the limitations of the current study. First, the information of household fuel use was self-reported; hence, the recall bias could not be avoided. Second, despite considering many covariates, we could not rule out the possibility that residual and unmeasured factors might have contributed to the association observed. For example, physical activity, sedentary behavior, indoor second-hand smoke exposures, frequency of household fuel use and heating/cookstove ventilation use were not considered in the present study because of inadequate data. We recommend more attention on handgrip strength changes due to household air pollution should paid especially on middle-aged and older adults in developing counties. Third, the results can only be generalized to middle-aged and older people in China, and the association between household fuel use and HGS may be different between young individuals or other countries. Forth, since many participants were excluded owing to missing data for main variables, this might have led to selection bias. Additional studies are warranted to verify our findings. Fifth, due to the limitation of data, the use of household fuel use for exposure classification (such as exposure of people who actually cooked with the fuel vs. those who could stay away from the stove/ kitchen) was not considered in our study. Moreover, because the outdoor air pollution could have affected the results, we adjusted for ambient concentration of PM2.5 in the regression models. The ambient concentration of PM2.5 was at the city-level but not individual-level because of lack of detailed address. Finally, we used traditional approach to evaluate HGS, new indicators (such as smart multifunction novel prototype dynamometer, named BodyGrip) (29) should be considered in future studies.

## Conclusion

The use of solid fuel for cooking but not heating was associated with greater decreases of HGS in middle-aged and older Chinese individuals. Household air pollution caused by indoor solid fuel combustion should be given attention to. The findings are in favor of the national policies to popularize the use of clean fuel to a certain degree.

## Data Availability Statement

Publicly available datasets were analyzed in this study. This data can be found here: The data that support the findings of this study are openly available in [China Health and Retirement Longitudinal Study] at [http://charls.pku.edu.cn/index.html], reference number.

## Ethics Statement

The studies involving human participants were reviewed and approved by The Ethical Committees of Peking University had approved the protocol of this analysis. All participants provided written informed consent. All study procedures were performed according to the ethical guidelines of the 1975 Declaration of Helsinki. The patients/participants provided their written informed consent to participate in this study.

## Author Contributions

Study concept and design: YZ, YX, and YL. Acquisition of data and drafting of the manuscript: YL and QC. Analysis and interpretation of data: YX and YL. Critical revision of the manuscript for important intellectual content: YZ and YX. All authors contributed to the article and approved the submitted version.

## Funding

This work was supported by the National Key R&D Program of China (No. 2017YFC0907404).

## Conflict of Interest

The authors declare that the research was conducted in the absence of any commercial or financial relationships that could be construed as a potential conflict of interest.

## Publisher's Note

All claims expressed in this article are solely those of the authors and do not necessarily represent those of their affiliated organizations, or those of the publisher, the editors and the reviewers. Any product that may be evaluated in this article, or claim that may be made by its manufacturer, is not guaranteed or endorsed by the publisher.

## Abbreviations

HGS: handgrip strength
PM: particulate matter
PM2.5: fine inhalable particles with diameters that are generally 2.5 micrometers and smaller
CHARLS: China Health and Retirement Longitudinal Study.
