# Supplementary material for: Longitudinal Associations Between Household Solid Fuel Use and Handgrip Strength in Middle-Aged and Older Chinese Individuals: The China Health and Retirement Longitudinal Study
Source: Front Public Health. 2022 Jun 30;10:881759. doi: 10.3389/fpubh.2022.881759 (PMC9280178; doi:10.3389/fpubh.2022.881759)
Supplement: Supplementary file 1 [file Data_Sheet_1.pdf]

Supplementary Material

Detailed information of assessment and the definition of confounding factors:

For further analysis, educational levels were classified into "no formal education", "primary school", or "middle school or above"; smoking status was classified as "current smoker", "ex-smoker", or "non-smoker"; drinking status was classified as "≥ 1 time/month", "< 1 time/month", or "never"; marital status was classified as "currently married" or not; place of residence was classified as "rural village" or "urban community"; income was classified as "≥ mean value" or not; participation in social activities was based on whether the individuals participated in social activities (such as interacted with friends; played Mah-jong, chess, cards, or visited a community-related organization; provided help to family, friends, or neighbors or cared for a sick or disabled adult who do not live with you and who did not pay you for the help; went to a sport, social, or other kind of club; completed volunteer or charity work; attended an educational or training course; invested in stock; used the Internet) in the last month; the number of chronic diseases was classified as "0", "1", or "≥ 2"; retirement status was classified as "retired" or not. Body mass index (BMI) was calculated as the weight in kilograms divided by the square of the height in meters (kg/m^2).
